# Why do inequality and deprivation produce high crime and low trust?

**DOI:** 10.1038/s41598-020-80897-8

**Published:** 2021-01-21

**Authors:** Benoît De Courson, Daniel Nettle

**Affiliations:** 1 Institut Jean Nicod, Département d’études cognitives, ENS, EHESS, PSL Research University, CNRS, Paris, France; 2 Population Health Sciences Institute, Newcastle University, Newcastle, UK

**Keywords:** Psychology, Human behaviour

## Abstract

Humans sometimes cooperate to mutual advantage, and sometimes exploit one another. In industrialised societies, the prevalence of exploitation, in the form of crime, is related to the distribution of economic resources: more unequal societies tend to have higher crime, as well as lower social trust. We created a model of cooperation and exploitation to explore why this should be. Distinctively, our model features a desperation threshold, a level of resources below which it is extremely damaging to fall. Agents do not belong to fixed types, but condition their behaviour on their current resource level and the behaviour in the population around them. We show that the optimal action for individuals who are close to the desperation threshold is to exploit others. This remains true even in the presence of severe and probable punishment for exploitation, since successful exploitation is the quickest route out of desperation, whereas being punished does not make already desperate states much worse. Simulated populations with a sufficiently unequal distribution of resources rapidly evolve an equilibrium of low trust and zero cooperation: desperate individuals try to exploit, and non-desperate individuals avoid interaction altogether. Making the distribution of resources more equal or increasing social mobility is generally effective in producing a high cooperation, high trust equilibrium; increasing punishment severity is not.

## Introduction

Humans are often described as an unusually cooperative or ‘ultrasocial’ species^1^. The truth is more complex: humans from the same society can cooperate for mutual benefit; or they can simply co-exist; or they can actively exploit one another, as in, for example, crime. A theory of human sociality should ideally predict what mix of these alternatives will emerge under which circumstances. Comparing across industrialised societies, higher inequality—greater dispersion in the distribution of economic resources across individuals—is associated with higher crime and lower social trust^2–7^. These associations appear empirically robust, and meet epidemiological criteria for being considered causal^8^. However, the nature of the causal mechanisms is still debated. The effects of inequality are macroscopic phenomena, seen most clearly by comparing aggregates such as countries or states. It is their micro-foundations in individual psychology and behaviour that still require clarification.

There are, broadly, two classes of explanation for how inequality, a population-level phenomenon, could influence individual-level outcomes like crime or trust. The first class of explanation is compositional: in more unequal societies, the least fortunate individuals are absolutely worse off than in more equal societies of the same average wealth, exactly because the dispersion either side of the average is greater. Some individuals are also better off too, at the other end of the distribution, but if there is any non-linearity in the function relating individual resources to outcomes—if for example the poor becoming absolutely poorer has a larger effect on their propensity to offend than the rich becoming absolutely richer has on theirs—this can still change outcome prevalence in the population^9–11^. In line with compositional explanations, across US counties, the association between inequality and rate of property crime is fully mediated by the prevalence of poverty, which is higher in more unequal counties^2^. Moreover, changes in rates over time track changes in the economic prospects of people at the bottom end of the socioeconomic distribution^12,13^. The second class of explanation is psychosocial: individuals perceive the magnitude of social differentials in the society around them, and this affects their state of mind, increasing competitiveness, anxiety and self-serving individualism^8,14^. In this paper, we develop an explanatory model for why greater inequality should produce higher crime and lower social trust. Our model provides a bridge between compositional and psychosocial explanations. Its explanation for the inequality-crime association is compositional: individuals offend when their own absolute level of resources is desperately low, and the effect of increasing inequality is to make such desperation more prevalent. On the other hand, the model’s explanation for the inequality-trust association is more psychosocial: all individuals in high-inequality populations end up trusting less, regardless of their personal resource levels.

To provide a micro-foundation in individual behaviour for the macro-level effects of inequality on crime, we must start from explanations for why individuals commit crimes. Economic^15,16^ and behavioural-ecological^17^ approaches see offending as a strategic response to specific patterns of incentive. Economic models predict that offending should be more attractive when the payoffs from legitimate activity are low. This principle successfully explains variation in offending behaviour both within and between societies^12,16^. It can also explain the relationship between crime levels and inequality, in compositional manner, because unequal societies produce poorer legitimate opportunities for people at the lower end of the socioeconomic spectrum^2^. However, these models are generally taken to predict that making punishments for crime more severe should reduce the prevalence of offending, because harsher punishment should reduce the expected utility associated with the criminal option. Empirical evidence, though, does not clearly support the hypothesis that increasing punishment severity reduces offending^18,19^. There is more evidence for a deterrent effect of increased *probability* of punishment, though even this effect may be modest^18,19^.

Becker^15^ pointed out that the puzzle of the weak deterrent effect of punishment severity would be solved if offenders were risk-preferring. The decision to offend is risky in that it has either a large positive payoff (if not caught) or a large negative one (if caught and punished). An individual who prefers risk might thus choose to offend even if the expected utility of offending is negative due to a possible severe punishment. Thus, the question becomes: why would some people—those who commit crime—prefer risk, when people are usually averse to it? To address this question, our model incorporates features of classic risk-sensitive foraging theory from behavioural ecology^20^ (for a review in the context of human behaviour, see Ref.^21^). Risk-sensitive foraging models incorporate a desperation threshold: a level of resources below which it is disastrous to fall, in the foraging case because of starvation. The models show that individuals in sufficient imminent danger of falling below this threshold ought to become risk-preferring. If a risky option is successful, it will allow them to leap back over the threshold; and if not, their prospects will be no more dire than they were anyway. Our model is novel in explicitly incorporating a desperation threshold into decisions about whether to cooperate (analogous in our model to participating in legitimate economic activity) or exploit others (analogous to committing an acquisitive crime).

The desperation threshold is the major theoretical innovation of our model. We justify its inclusion on multiple grounds. First, the ultimate currency in our model is fitness, a quantity with a natural biological interpretation that must necessarily be zero if the individual lacks the minimal resources to subsist and function socially. Thus, it is reasonable that expected fitness should be related to resource levels, but not linearly: there should be a point where, as resources deplete, expected fitness rapidly declines to zero. Our threshold assumption produces exactly this type of function (see Supplementary Sect. 2.1, Supplementary Fig. S1). Second, in experimental games where gaining a payoff is subject to a threshold, people do switch to risk-proneness when in danger of falling below the threshold, as risk-sensitive foraging theory predicts^22^. Although this does not show that such thresholds are widespread or important in real life, it does show that people intuitively understand their implications when they are faced with them, and respond accordingly. Third, there are ethnographic descriptions of ‘disaster levels’, ‘crisis levels’, or ‘edges’ that affect the risk attitudes of people facing poverty^23,24^. For example, writing on Southeast Asia, Scott^23^ describes the spectre of a “subsistence crisis level—perhaps a ‘danger zone’ rather than a ‘level’ would be more accurate…a threshold below which the qualitative deterioration in subsistence, security and status is massive and painful” (p. 17), as an ever-present factor in people’s decisions. Thus, including a desperation threshold is a simple but potentially powerful innovation into models of cooperation and exploitation, with potential to generate new insights.

In our model, agents repeatedly decide between three actions: foraging alone, foraging cooperatively, or exploiting a cooperative group. Foraging cooperatively is analogous to legitimate economic activity, and exploitation is analgous to acquisitive crime. Agents have variable levels of resources, and their behaviour is state-dependent. That is, rather than having a fixed strategy of always cooperating or always exploiting, each agent, at each interaction, selects a behaviour based on their current level of resources, the behaviour of others in the surrounding population, and background parameters such as the probability and severity of punishment, and the likelihood of resources improving through other means. Agents seek to maximize fitness. We assume that fitness is positively related to resource levels, but that there is a threshold, a critically low level of resources below which there is an immediate fitness penalty for falling. Our investigation of the model has two stages. We first compute the optimal action policy an individual should follow; that is, the optimal action to select for every possible combination of the situational variables. Second, we simulate populations of individuals all following the optimal action policies, to predict population-level outcomes for different initial resource distributions.

To explain the model in more detail, at each time point *t* in an indefinitely long sequence of time steps (where one time step is one economic interaction), agents have a current level of resources *s.* They can take one of three actions. *Foraging alone* costs *x* units of resources and is also guaranteed to return *x.* Thus, foraging alone is sufficient to maintain the agent but creates no increase in resources. It is also safe from exploitation, as we conceptualise it as involving minimal interaction with others. Alternatively, agents can team up with *n-1* others to *cooperate*. As long as no other group member exploits, cooperation is mutually beneficial, costing *x* units but producing a payoff of $\alpha x \left( {\alpha > 1} \right)$ to each group member. Finally, agents can *exploit*: join a cooperating group and try to selfishly divert the resources produced therein. If this exploitation is successful, they obtain a large reward β, but if they fail, they receive a punishment π. The probability of being punished is γ. The punishment is not administered by peers: we assume that there is a central punitive institution in place, and both the size and probability of punishment are exogenous. In our default case, the expected payoff for exploitation is zero (i.e. $\left( {1 - \gamma } \right)\beta = \gamma \pi$), making exploitation no better than foraging alone on average, and worse than cooperating. However, the reward for a successful exploitation, β, is the largest payoff available to the agent in any single time step.

At every time step, each agent’s resource level is updated according to the outcomes of their action. In addition, resource levels change by a disturbance term controlled by a parameter *r*, such that the mean and variance of population resources are unchanged, but the temporal autocorrelation of agents’ resource levels is only 1 − *r*. If *r* is high, individuals whose current resources are low can expect they will be higher in the future and vice versa, because of regression to the mean. If $r = 0$, resources will never change other than by the agent’s actions. We consider *r* a measure of social mobility due to causes other than choice of actions.

In the first stage, we use stochastic dynamic programming^25,26^ to compute the optimal action policy. Fitness is a positive linear function of expected resource level *s* in the future. However, in computing the fitness payoffs of each action, we also penalize, by a fixed amount, any action that leaves the agent below a desperation threshold in the next time step (arbitrarily, we set this threshold at *s* = 0). The optimal action policy identifies which one of the three actions is favoured for every possible combination of the factors that impinge on the agent. These include both their own current resource state *s*, and features of their social world, such as the severity of punishment π, the probability of punishment γ, and the level of social mobility *r.* A critical variable that enters into the computation of the optimal action is the probability that any cooperating group in the population will contain someone who exploits. We denote this probability *p*. We can think of 1 − *p* as an index of the trustworthiness of the surrounding population. Computing the optimal policy effectively allows us to ask: under what circumstances *should* an individual forage alone, cooperate, or exploit?

In the second stage, we simulate populations of agents all following the optimal policies computed in the first stage. We can vary the starting distributions of resources (their mean and dispersion), as well as other parameters such as social mobility and the probability and severity of punishment. During the simulation stage, each agent forms an estimate of 1 − *p*, the trustworthiness of others, through observing the behaviour of a randomly-selected subset of other individuals. We refer to these estimates as the agents’ *social trust,* since social trust is defined as the generalized expectation that others will behave well^27^. Social trust updates at the end of each time step. Agents’ social trust values are unbiased estimates of the current trustworthiness of the surrounding population, but they are not precise, because they are based on only a finite sample of other population members. The simulation stage, allows us to ask: what are the predicted temporal dynamics of behaviour, and of social trust, in populations with different starting distributions of resources, different levels of social mobility, and different punishments for exploitation?

## Results

**Figure 1:**
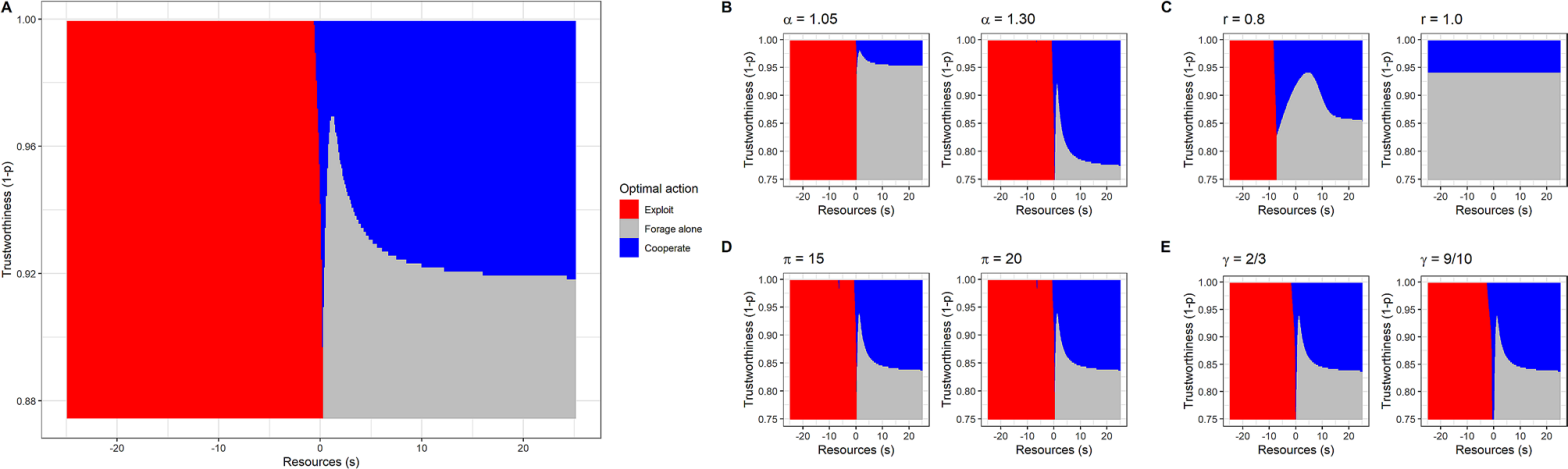
Optimal actions as a function of the individual’s current resources *s* and the trustworthiness of the surrounding population, 1 − *p*. (**A**) All parameters at their default values. This includes: α = 1.2, r = 0.1, π = 10, and γ = 1/3 (see Table 1 for a full list). (**B**) Effect of altering the efficiency of cooperation α to be either lower (1.05) or higher (1.30) than (**A**). Other parameter values are as for (**A**). (**C**) Effects of varying social mobility, to be either high (r = 0.8), or complete (r = 1.0; i.e. resource levels in this time period have no continuity at all into the next). Other parameter values are as for (**A**). (**D**) Effect of increasing the severity of punishment for exploiters to π = 15 and π = 20. Other parameter values are as for (**A**). (**E**). Effects of altering the probability of punishment for exploiters to γ = 2/3 and γ = 9/10. Other parameter values are as for (**A**).

Each of the three actions is optimal in a different region of the space formed by current resources *s* and the trustworthiness of others 1 − *p* (Fig. 1a). Below a critical value of *s*, agents should always exploit, regardless of trustworthiness*.* In the default case, this critical value is in the vicinity of the desperation threshold, though it can be lower or higher depending on the value of other parameters. With our default values, exploitation will not, on average, make the agent’s resource state any better in subsequent time steps. However, there is a large advantage to getting above the threshold in the next time step, and there is a region of the resource continuum where exploitation is the only action that can achieve this in one go (intuitively, it is the quickest way to ‘get one’s head above water’). Where *s* is above the critical value, cooperation is optimal as long as the trustworthiness of the surrounding population is sufficiently high. However, if trustworthiness is too low, the likelihood of getting exploited makes cooperation worse than foraging alone. The shape of the frontier between cooperation and foraging alone is complex when resources are close to the desperation threshold. This is because cooperation and foraging alone also differ in riskiness; foraging alone is risk-free, but cooperation carries a risk of being exploited that depends on trustworthiness. Just above the exploitation zone, there is a small region where cooperation is favoured even at low trustworthiness, since one successful cooperation would be enough to hurdle back over the threshold, but foraging alone would not. Just above this is a zone where foraging alone is favoured even at high trustworthiness; here the agent will be above the threshold in the next time period unless they are a victim of exploitation, which makes them averse to taking the risk of cooperating.

We explored the sensitivity of the optimal policy to changes in parameter values. Increasing the profitability of cooperation (α) decreases the level of trustworthiness that is required for cooperation to be worthwhile (Fig. 1B; analytically, the cooperation/foraging alone frontier for $s \gg 0$ is at $\left( {1 - p} \right) = 1/\alpha$; see Supplementary Sect. 2.2). A very high level of social mobility *r* moves the critical value for exploitation far to the left (i.e. individuals have to be in an even more dire state before they start to exploit; Fig. 1C). This is because with high social mobility, badly-off individuals can expect that their level of resources will regress towards the mean over time anyway, lessening the need for risky action when faced with a small immediate shortfall.

The optimality of exploitation below the critical level of resources is generally insensitive to increasing the severity of punishment, π (Fig. 1D), even where the expected value of exploitation is thereby rendered negative. This is because a desperate agent will be below the threshold in the next time step anyway if they forage alone, cooperate, or receive a punishment of any size. They are so badly off that it is relatively unimportant how much worse things get, but important to take any small chance of ‘jumping over’ the threshold. The exploitation boundary is slightly more sensitive to the probability of punishment, γ, though even this sensitivity is modest (Fig. 1E). When γ is very high, it is optimal for agents very close to the boundary of desperation to take a gamble on cooperating, even where trustworthiness is rather low. Although this is risky, it offers a better chance of getting back above the threshold than exploitation that is almost bound to fail. Nonetheless, it is striking that even where exploitation is almost bound to fail and attracts a heavy penalty, it is still the best option for an individual whose current resource level is desperately low.

We also explored the effect of setting either the probability γ or the severity π of punishment so low that the expected payoff from exploitation is positive. This produces a pattern where exploitation is optimal if an agent’s resources are either desperately low, or comfortably high (see Supplementary Fig. S2). Only in the middle—currently above the threshold, but not by far enough that a punishment would not pull them down below it–should agents cooperate or forage alone.

**Figure 2:**
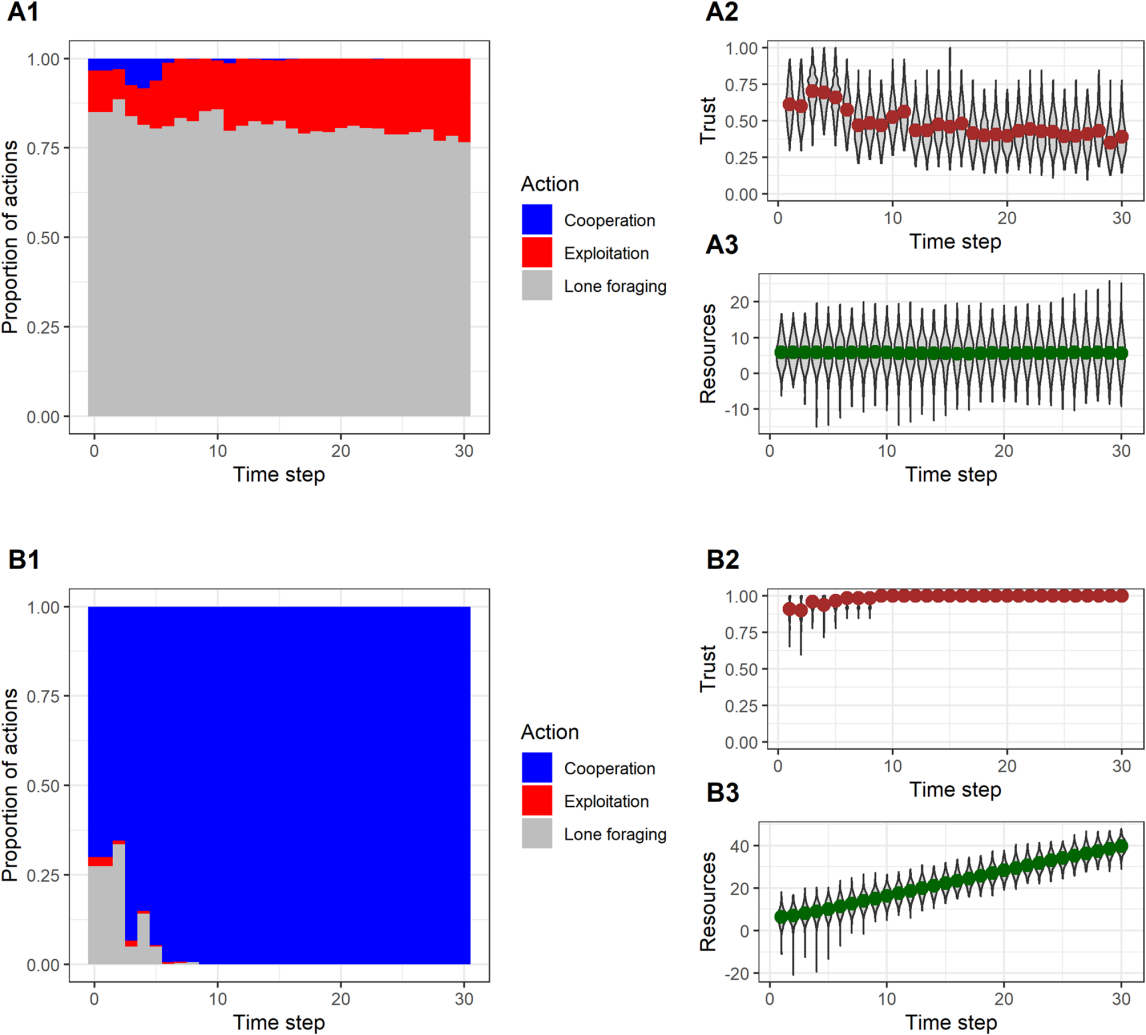
The two equilibria in simulated populations. (**A**) The poverty trap. There is sufficient exploitation in the first time step (**A1**) that social trust is low (**A2**). Consequently, potential cooperators switch to lone foraging, resources never increase (**A3**), and a subgroup of the population is left below the threshold seeking to exploit. Simulation initialised with μ = 5.5, σ = 4 and all other parameters at their default values. (**B**) The virtuous circle. Exploitation is sufficiently rare from the outset (**B1**) that trust is high (**B2**) and individuals switch from lone foraging to cooperation. This drives an increase in resources, eventually lifting almost all individuals above the threshold. Simulation initialised with μ = 5.5, σ = 3 and all other parameters at their default values.

We simulated populations of *N* = 500 individuals each following the optimal policy, with the distribution of initial resources *s* drawn from a distribution with mean μ and standard deviation σ. Populations fall into one of two absorbing equilibria. In the first, *the poverty trap* (Fig. 2A), there is no cooperation after the first few time periods. Instead, there is a balance of attempted exploitation and foraging alone, with the proportions of these determined by the initial resource distribution and the values of π and γ. The way this equilibrium develops is as follows: there is a sufficiently high frequency of exploitation in the first round (about 10% of the population or more is required) that subsequent social trust estimates are mostly very low. With trust low, those with the higher resource levels switch to foraging alone, whilst those whose resources are desperately low continue to try to exploit. Since foraging alone produces no surplus, the population mean resources never increases, and both exploiters and lone foragers are stuck where they were.

In the second equilibrium, the *virtuous circle* (Fig. 2B), the frequency of exploitation is lower at the outset. Individuals whose resources are high form high assessments of social trust, and hence choose cooperation over foraging alone. Since cooperation creates a surplus, the mean level of resources in the population increases. This benefits the few exploiters, both through the upward drift of social mobility, and because they sometimes exploit successfully. This resolves the problem of exploitation, since in so doing they move above the critical value to the point where it is no longer in their interests to exploit, and since they are in such a high-trust population, they then start to cooperate. Thus, over time, trust becomes universally high, resources grow, and cooperation becomes almost universal.

Each of the two equilibria has a basin of attraction in the space of initial population characteristics. The poverty trap is reached if the fraction of individuals whose resource levels fall below the level that triggers exploitation is sufficiently large at any point. With the desperation threshold at s = 0, his fraction is affected by both the mean resources μ, and inequality σ. For a given μ, increasing σ (i.e. greater inequality) makes it more likely that the poverty trap will result, because, by broadening the resource distribution, the tail that protrudes into the desperation zone is necessarily made larger.

**Figure 3:**
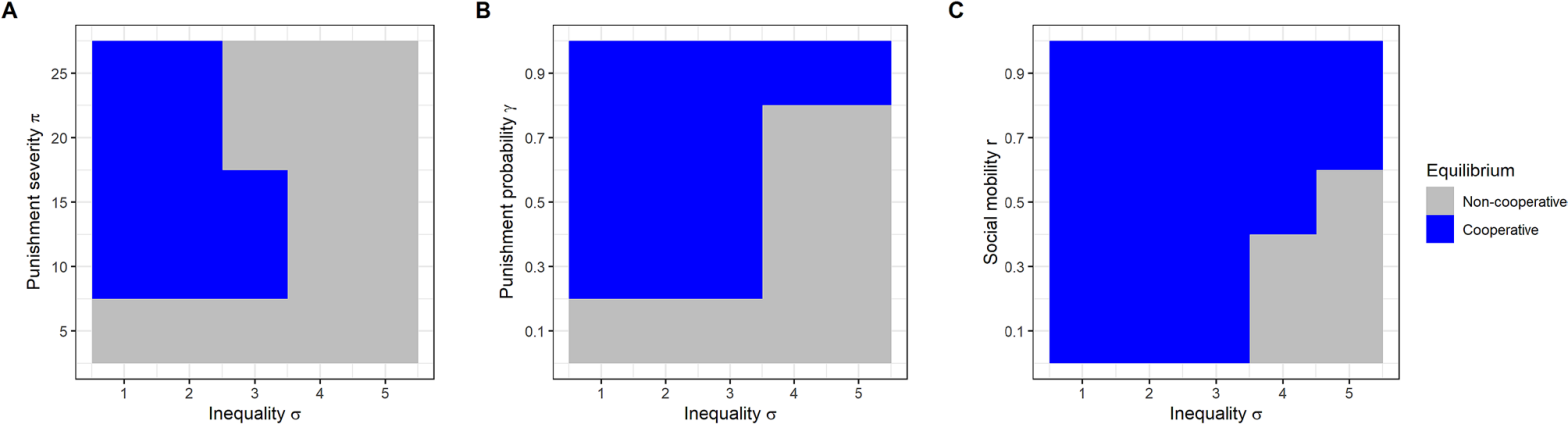
Equilibrium population states by starting parameters. (**A**) Varying the initial inequality in resources σ and the severity of punishment π, whilst holding constant the probability of punishment γ at 1/3 and social mobility *r* at 0.1. (**B**) Varying the initial inequality in resources σ and the probability of punishment γ whilst holding the severity of punishment constant at π = 10 and social mobility *r* at 0.1. (**C**) Varying the initial inequality in resources σ and the level of social mobility *r* whilst holding constant the probability of punishment γ at 1/3 and the severity of punishment π at 10.

The boundaries of the basin of attraction of the poverty trap are also affected by severity of punishment, probability of punishment, and the level of social mobility (Fig. 3). If the severity of punishment π is close to zero, there is no disincentive to exploit, and the poverty trap always results. As long as a minimum size of punishment is met, further increases in punishment severity have no benefit in preventing the poverty trap (Fig. 3A). Indeed, there are circumstances where more severe punishment can make things worse. When the population has a degree of initial inequality that puts it close to the boundary between the two equilibria, very severe punishment (π = 20 or π = 25) pushes it into the poverty trap. This is because any individual that once tries exploitation because they are close to threshold (and is unsuccessful) is pushed so far down in resources by the punishment that they must then continue to exploit forever. Increasing the probability of punishment γ does not have this negative effect (Fig. 3B). Instead, a very high probability of punishment can forestall the poverty trap at levels of inequality where it would otherwise occur, because it causes some of the worst-off individuals to try cooperating instead, as shown in Fig. 1E. Finally, very high levels of social mobility *r* can rescue populations from the poverty trap even at high levels of inequality (Fig. 3C). This is because of its dramatic effect on the critical value at which individuals start to exploit, as shown in Fig. 1C.

Though the equilibria are self-perpetuating without exogenous forces, the system is highly responsive to shocks. For example, exogenously changing the level of inequality in the population (via imposing a reduction in σ after 16 time steps) produces a phase transition from the poverty trap to the virtuous circle (Supplementary Fig. S3). This change is not instantaneous. First, a few individuals cross the threshold and change from exploitation to foraging alone; this produces a consequent change in social trust; which then leads to a mass switch to cooperation, and growth in mean wealth.

Results so far are all based on cooperation occurring in groups of size *n* = 5. Reducing *n* enlarges the basin of attraction of the virtuous circle (Supplementary Sect. 2.5, Supplementary Fig. S4). This is because, for any given population prevalence of exploitation, there is more likely to be at least one exploiter in a group of five than a group of three. Reducing the interaction group size changes the trustworthiness boundary between the region where it is optimal to cooperate and the region where it is better to forage alone. Thus, there are parameter values in our model where populations would succumb to the poverty trap by attempting to mount large cooperation groups, but avoid it by restricting cooperation groups to a smaller size.

## Discussion

In our model, exploiting others can be an individual’s optimal strategy under certain circumstances, namely when their resource levels are very low, and cannot be expected to spontaneously improve. We extend previous models by showing that it can be optimal to exploit even when the punishment for doing so and being caught is large enough to make the expected utility of exploitation negative. Two conditions combine to make this the case. First, exploitation produces a large variance in payoffs: it is costly to exploit and be caught, but there is a chance of securing a large positive payoff. Second, there is a threshold of desperation below which it is extremely costly to fall. It is precisely when at risk of falling below this threshold that exploitation becomes worthwhile: if it succeeds, one hurdles the threshold, and if it fails, one is scarcely worse off than one would have been anyway. In effect, due to the threshold, there is a point where agents have little left to lose, and this makes them risk-preferring. Thus, our model results connect classic economic models of crime^15,16^ to risk-sensitive foraging theory from behavioural ecology^20^. In the process, it provides a simple answer to the question that has puzzled a number of authors^18,19^: why aren’t increases in the severity of punishments as deterrent as simple expected utility considerations imply they ought to be? Our model suggests that, beyond a minimum required level of punishment, not only might increasing severity be ineffective at reducing exploitation. It could under some circumstances make exploitation worse, by pushing punishees into such a low resource state that they have no reasonable option but to continue exploiting. Our findings also have implications for the literature on the evolution of cooperation. This has shown that punishment can be an effective mechanism for stabilising cooperation^28,29^, but have not considered that the deterrent effects of punishment may be different for different individuals, due to variation in their states. Our findings could be relevant to understanding why some level of exploitation persists in practice even when punishment is deterrent overall.

Within criminology, our prediction of risky exploitative behaviour when in danger of falling below a threshold of desperation is reminiscent of Merton’s strain theory of deviance^30,31^. Under this theory, deviance results when individuals have a goal (remaining constantly above the threshold of participation in society), but the available legitimate means are insufficient to get them there (neither foraging alone nor cooperation has a large enough one-time payoff). They thus turn to risky alternatives, despite the drawbacks of these (see also Ref.^32^ for similar arguments). This explanation is not reducible to desperation making individuals discount the future more steeply, which is often invoked as an explanation for criminality^33^. Agents in our model do not face choices between smaller-sooner and larger-later rewards; the payoff for exploitation is immediate, whether successful or unsuccessful. Also note the philosophical differences between our approach and ‘self-control’ styles of explanation^34^. Those approaches see offending as deficient decision-making: it would be in people’s interests not to offend, but some can’t manage it (see Ref.^35^ for a critical review). Like economic^15,16^ and behavioural-ecological^17^ theories of crime more generally, ours assumes instead that there are certain situations or states where offending is the best of a bad set of available options.

As well as a large class of circumstances where only individuals in a poor resource state will choose to exploit, we also identify some—where the expected payoff for exploitation is positive—where individuals with *both* very low and very high resources exploit, whilst those in the middle avoid doing so. Such cases have been anticipated in theories of human risk-sensitivity^21^. These distinguish risk-preference through need (e.g. to get back above the threshold immediately) from risk-preference through ability (e.g. to absorb a punishment with no ill effects), predicting that both can occur under some circumstances^32^. This dual form of risk-taking is best analogised to a situation where punishments take the form of fines: those who are desperate have to run the risk of incurring them, even though they can ill afford it; whilst those who are extremely well off can simply afford to pay them if caught. When we simulate populations of agents all following the optimal strategies identified by the model, population-level characteristics (inequality of resources, level of social mobility) affect the prevalence of exploitation and the level of trust. Specifically, holding constant the average level of resources, greater inequality makes frequent exploitation and low trust a more likely outcome. Thus, we capture the widely-observed associations between inequality, trust and crime levels that were our starting point^2–6^. Note that our explanation for the inequality-crime nexus is basically compositional rather than psychosocial. Decisions to offend are based primarily on agents’ own levels of resources; these are just more likely to be desperately low in more unequal populations. Turning these simulation findings into empirical predictions, we would expect the association between inequality and crime rates to be driven by more unequal societies producing worse prospects for people at the bottom end of the resources distribution, who would be the ones who turn to property crime. Inequality effects at the aggregate level should be largely mediated by individual-level poverty. There is evidence compatible with these claims for property crime^2,12,13^. This is the type of crime most similar to our modelled situation. Non-acquisitive crimes of violence, though related to inequality, do not appear so strongly mediated by individual-level poverty, and may thus require different but related explanations^2,36^.

However, the other major result of our population simulations—that more unequal populations are more likely to produce low trust—is not compositional. In our unequal simulated populations, *every* agent has low trust, not just the ones at the bottom of the resource distribution. This is compatible with empirical evidence: the association between inequality and social trust survives controlling for individual poverty^6^. Thus, our model generates a genuinely ecological effect of inequality on social relationships that fits the available evidence and links it to the psychosocial tradition of explanation^37^. Indeed, the model suggests a reason why psychosocial effects should arise. For agents above the threshold, the optimal decision between cooperation and foraging alone depends on inferences about whether anyone else in the population will exploit. To know that, you have to attend to the behaviour of everyone else, not just your own state. Thus, the model naturally generates a reason for agents to be sensitive to the distribution of others’ states in the population (or at the very least their behaviour), and to condition their social engagement with others on it.

In as much as our model provides a compositional explanation for the inequality-crime relationship, it might seem to imply that high levels of inequality would not lead to high crime as long as the mean wealth of the population was sufficiently high. This is because, with high mean wealth, even those in the bottom tail of the distribution would have sufficient levels of resources to be above the threshold of desperation. However, this implication would only follow if the location of the desperation threshold is considered exogenous and fixed. If, instead, the location of the desperation threshold moves upwards with mean wealth of the population, then more inequality will always produce more acquisitive crime, regardless of the mean level of population wealth. Assuming that the threshold moves in this way is a reasonable move: definitions of poverty for developed countries are expressed in terms of the resources required to live a life seen as acceptable or normal within that society, not an absolute dollar value (see Ref.^36^, pp. 64–6). Moreover, there is clear evidence that people compare themselves to relevant others in assessing the adequacy of their resources^38^. Thus, we would expect inequality to remain important for crime regardless of overall economic growth.

In addition to the results concerning inequality, we found that social mobility should, other things being equal, reduce the prevalence of exploitation, although social mobility has to be very high for the effect to be substantial. The pattern can again be interpreted as consistent with Merton’s strain theory of deviance^31^: very high levels of social mobility provide legitimate routes for those whose state is poor to improve it, thus reducing the zone where deviance is required. Economists have noted that those places within the USA with higher levels of intergenerational social mobility also have lower crime rates^39,40^. Their account of the causality in this association is the reverse of ours: the presence of crime, particularly violent crime, inhibits upward mobility^39^. However, it is possible that social mobility and crime are mutually causative.

Like any model, ours simplifies social situations to very bare elements. Interaction groups are drawn randomly at every time step from the whole population. Thus, there are no ongoing personal relationships, no reputation, no social networks, no kinship, no segregation or assortment of sub-groups. The model best captures social groups with frequent new interactions between strangers, which is appropriate since the phenomena under investigated are documented for commercial and industrial societies. A problem in mapping our findings onto empirical reality is that our population simulations generate two discrete equilibria: zero trust, economic stagnation and zero cooperation, or almost perfect trust, unlimited economic growth and zero exploitation. Although we show that the distribution of resources determines which equilibrium is reached, our model as presented here does generate the continuous relationships between inequality, crime, and trust (or indeed inequality and economic growth^41^) that have been observed in reality. Even the most unequal real society features some social cooperation, and even the most equal features some property crime; the effects of inequality are graded. We make two points to try to bridge the disconnect between the black and white world of the simulations and the shades of grey seen in reality. First, our model *does* predict a continuous relationship between the level of inequality and the maximum size of cooperating groups. A highly unequal population, containing many individuals with an incentive to exploit, might only be able to sustain collective actions at the level of a few individuals, whereas a more equal population where almost no-one has an incentive to exploit could sustain far larger ones. Second, we appeal to all the richness of real social processes that our model excludes. In unequal countries, although social trust is relatively low, people can draw more heavily on their established social networks and reputational information; more homogenous sub-groups can segregate themselves; people can use defensive security measures, to keep cooperative relationships ongoing and protected; and so forth. Investment in these kinds of measures may vary proportionately with inequality and trust, thus maintaining outcomes intermediate between the stark equilibria of our simulations. Our key findings also depend entirely on accepting the notion that there is a threshold of desperation, a substantial non-linearity in the value of having resources. As we outlined in the Introduction, we believe there are good grounds for exploring the implications of such an assumption. However, that is very different from claiming that the widespread existence of such thresholds has been demonstrated. We hope our findings might generate empirical investigation into both the objective reality and psychological appraisal of such thresholds for people in poverty.

Limitations and simplifications duly noted, our model does have some clear implications. Large population-scale reductions in crime and exploitation should not be expected to follow from increasing the severity of punishments, and these could conceivably be counterproductive. Addressing basic distributional issues that leave large numbers of people in desperate circumstances and without legitimate means to improve them will have a much greater effect. Natural-experimental evidence supports this. The Eastern Cherokee, a Native American group with a high rate of poverty, distributed casino royalties through an unconditional income scheme. Rates of minor offending amongst young people in recipient households decline markedly, with no changes to the judicial regime^42^. Improving the distribution of resources would also be expected to increase social trust, and with it, the quality of human relationships; and this, for everyone, not just those in desperate circumstances.

## Methods

The model was written in Python and implemented via a Jupyter notebook. For a fuller description of the model, see Supplementary Sect. 1 and Supplementary Table S1.

### Computing optimal policies

We used a stochastic dynamic programming algorithm^25,26^. Agents choose among a set of possible actions, defined by (probabilistic) consequences for the agent’s level of resources *s*. We seek, for every possible value of *s* and of *p* the agent might face, and given the values of other parameters, the action that maximises expected fitness. Maximization is achieved through backward induction: we begin with a ‘last time step’ (*T*) where terminal fitness is defined, as an increasing linear function of resource level *s*. Then in the period *T* − 1 we compute for each combination of state variables and action the expected fitness at *T*, and thus choose for the optimal action for every combination of states. This allows us define expected fitness for every value of the state variables at *T* − 1, repeat the maximization for time step *T* − 2, and so on iteratively. The desperation threshold is implemented as a fixed fitness penalty *ω* that is applied whenever the individual’s resources are below the threshold level *s* = 0. As the calculation moves backwards away from *T*, the resulting mapping of state variables to optimal actions converges to a long term optimal policy.

### Actions and payoffs

Agents choose among three actions:

- *Cooperate* The agent invests *x* units of resource and is rewarded *α*·*x* with probability 1 − *p* (*p* is the probability of cooperation being exploited, and 1 − *p* is therefore the trustworthiness of the surrounding population), and 0 with probability *p*. The net payoff is therefore *x* · (*α* − 1) if there is no exploitation and − *x* if there is. We assume that *α* > 1 (by default *α* = 1*.*2), which means that cooperation is more efficient than foraging alone. For the computation of optimal policies, we treat *p* as an exogenous variable. In the population simulations, it becomes endogenous.
- *Exploit* An agent joins a cooperating group, but does not invest x, and instead tries to steal their partners’ investments, leading to a reward of *β* if the exploitation succeeds and a cost *π* if it fails. The probability of exploitation failing (i.e. being punished) is *γ*.
- *Forage alone* The agent forages alone, investing *x* units of resource, receiving *x* in return, and suffering no risk of exploitation.

Payoffs are also affected by a random perturbation, so the above-mentioned payoffs are just the expected values. A simple form such as the addition of $\varepsilon \sim N\left( {0, \sigma^{2} } \right)$ would be unsuitable when used in population simulations. As the variance of independent random variables is additive, it would lead to an ever increasing dispersion of resource levels in the population. To avoid this issue, we adopted a perturbation in the form of a first-order autoregressive process that does not change either the mean or the variance of resources in the population^43^:

$$ s_{t + 1} = \left( {1 - r} \right) \cdot s_{t} + r \cdot \varepsilon $$

$$ \varepsilon \sim N\left( {\mu , \frac{{1 - r^{2} }}{{\left( {1 - r} \right)^{2} }} \cdot \sigma^{2} } \right) $$

Here, *µ* is the current mean resources in the population and *σ*^2^ the population variance. The term $\left( {1 - r} \right) \in \left[ {0, 1} \right]$ represents the desired correlation between an agent’s current and subsequent resources, which leads to us describing *r* as the ‘social mobility’ of the population. The perturbation can be seen as a ‘shuffle’. Each agent’s resource level is attracted to *µ* with a strength depending on *r*, but this regression to the mean is exactly offset at the population level by the variance added by the perturbation, so that the overall distribution of resources is roughly unchanged. If *r* = 1, current resources are not informative about future resources.

### The dynamic programming equation

Let *I* be the set of actions (*cooperate*, *exploit* and *alone*), which we shorten as *I* = {*C,H,A*}. For *i* ∈ *I*, we denote as $\phi_{t}^{i} \left( {s, .} \right)$ the probability density of resources in in time step *t* if, in time step *t* − 1, the resource level is *s* and the chosen action *i*. The expressions of these functions were obtained through the law of total probability, conditioning on the possible outcomes of the actions (e.g. success or failure of exploitation and cooperation), and with the Gaussian density of the random variable.

We can now write the dynamic programming equation, which gives the backward recurrence relation to compute the payoff values (and the decisions) at the period *t* from the ones at the period *t* + 1.

$$ \begin{aligned} f_{t} \left( {s, p} \right) & = \mathop {\max }\limits_{i \in I} E_{i} (f_{t} + 1) \\ & = \mathop {\max }\limits_{i \in I} \smallint (f_{t + 1} \left( x \right) - \omega \cdot 1_{x < 0} ) \cdot \phi_{t + 1}^{i} \left( {s, p, x} \right)dx. \\ \end{aligned} $$

Here, $E_{i}$ is the conditional expectation if action *i* is played. The optimal action for the time step *t* is ${\text{argmax}}_{i \in I} E_{i} (f_{t} )$. The resource variable *s* was bounded in the interval [− 50, 50], and discretized with 1001 steps of size 0*.*1.

For any given set of parameters (summarised in Table 1), we can therefore compute the optimal decision rule. Note that we can distinguish two types of parameters:

- ‘Structural parameters’, i.e. those defining the ‘rules’ of the game (the payoffs for the actions and the level of social mobility *r*, for example). In the subsequent simulation phase, these parameters will be fixed for any run of the simulations.
- ‘Input parameters’, such as *p* and *s*. In the simulation phase, these will evolve endogenously.

**Table 1:** Main parameters used in modelling and simulations, with default values for structural parameters where appropriate.

Symbol	Meaning	Typical values
**Structural parameters**		
*r*	Social mobility	0.1
*x*	Cost of foraging	1
ω	Fitness cost below threshold	5
α	Efficiency of cooperation	1.2
β	Benefit of successful exploitation	5
π	Punishment for exploitation	10
γ	Probability of punishment	1/3
*T*	Total number of time steps	50
*N*	Population size	500
*n*	Cooperation group size	5
**Input parameters**		
μ	Population mean resources	
$\sigma^{2}$	Population variance resources	
*p*	Probability of being exploited	
*s*	Current level of resources	

Optimal policies rapidly stabilize as the computation moves away from *T*. We report optimal actions at *t* = 1 as the globally optimal actions.

### Population simulations

We begin each simulation by initializing a population of *N* = 500 individuals, whose resource levels are randomly drawn from a Gaussian distribution with a given mean *µ* and variance *σ*^2^. At each time step, interaction groups of *n* = 5 individuals are formed at random, and re-formed at each time step to avoid effects of assortment. There is no spatial structure in the populations. Each individual always follows the optimal policy for its resources *s* and its estimate of *p* (see below). Varying *N* has no effect as long as *N* > *n* and 500 is simply chosen for computational convenience.

To deal with the case where several members of the same interaction group choose to exploit, we choose one at random that exploits, and the others are deemed to forage alone (in effect, there is nothing left for them to take). Also, when there is no cooperator in the group, all exploiters are deemed to forage alone.

Rather than providing each individual with perfect knowledge of the trustworthiness of the rest of the population 1 − *p*, we allow individuals to form an estimate (their *social trust*) from their experience. Social trust is derived in the following way. Each agent observes the decision of a sample of *K* individuals in the population, counts the number *k* of exploiters and infers an (unbiased) estimate of the prevalence of exploiters in the population: $k^{\prime} = \frac{k}{K}N$ (rounded). The size of the sample can be varied to alter the precision with which agents can estimate trustworthiness. Unless otherwise stated we used *K* = 50. Since *p* is the probability that there will be at least one exploiter in an interaction group, it is one minus the probability that there will be zero exploiters. Each agent computes this from their *k’* by combinatorics.

An intentional consequence of social trust being estimated through sampling is that there is some population heterogeneity in social trust, and therefore in decisions about which action to take, even for agents with the same resources *s*. Note also that agents infer trustworthiness not from observing the particular individuals in their current interaction group, but rather, from a cross-section of the entire population. Thus, the estimate is genuinely *social trust* (the perception that people in society generally do or do not behave well).

## Supplementary Information

Supplementary Information.

## Data Availability

The Jupyter notebook for running the model is available at: https://github.com/regicid/Deprivation-antisociality. This repository also contains R code and datafiles used to make the figures in the paper.
