# Supplementary material for: Why do inequality and deprivation produce high crime and low trust?
Source: Sci Rep. 2021 Jan 21;11:1937. doi: 10.1038/s41598-020-80897-8 (PMC7820585; doi:10.1038/s41598-020-80897-8)
Supplement: Supplementary file 1 — Supplementary Information. [file 41598_2020_80897_MOESM1_ESM.pdf]

Supporting information for de Courson and Nettle, 'Why do deprivation and inequality produce high crime and low trust?'

Benoit de Courson and Daniel Nettle

November 10, 2020

1 Description of model

The model was written in Python and implemented via a Jupyter notebook. Code is available at: `https://github.com/regicid/Deprivation-antisociality`. We recommend using the version 'didactic-code.ipynb' for a simple way of getting started with the model. The repository also contains R code used to make the figures in the paper.

1.1 Computing optimal policies

We used a stochastic dynamic programming algorithm (Mangel and Clark 1988, Houston and McNamara 1999). Agents choose among a set of possible actions, defined by (probabilistic) consequences for the agent's level of resources s. We seek, for every value of s and of p (the probability that cooperation attempts will be exploited), the action that maximises expected fitness. Maximization is achieved through backward induction: we begin with a 'last time step' (T) where fitness is defined, as an increasing linear function of resource level s. Then in the period $T-1$ we compute for each combination of state variables and each action the expected fitness at T, and thus choose for the optimal action for every combination. This allows us define expected fitness for every value of the state variables at $T-1$, repeat the maximization for time step $T-2$, and so on iteratively. The desperation threshold is implemented as a fixed fitness penalty ω that is applied whenever the individual's resources are below the threshold level $s=0$. As the calculation moves backwards away from T, the resulting mapping of state variables to optimal actions converges to a long term optimal policy.

1.1.1 Actions and payoffs

Agents choose among three actions:

- *Cooperate*: The agent invests x units of resource and is rewarded $\alpha \cdot x$ with probability $1 - p$ (p is the probability of cooperation being exploited, and $1 - p$ is therefore the trustworthiness of the surrounding population), and 0 with probability p. The net payoff is therefore $x \cdot (\alpha - 1)$ if there is no exploitation and $-x$ if there is. We assume that $\alpha > 1$ (by default $\alpha = 1.2$), which means that cooperation is more efficient than foraging alone. For the computation of optimal policies, we treat p as an exogenous variable. In the population simulations, it becomes endogenous.
- *Exploit*: an agent tries to steal their partners' investments, leading to a reward of $\beta \cdot x$ if the exploitation succeeds and a cost π if it fails. the probability of exploitation success is γ. We consider cases where $(1-\gamma)\pi \geq \gamma\beta x$ in order that exploitation has a negative expected payoff.
- *Forage alone*: The agent forages alone, investing x units of resource, receiving x in return, and suffering no risk of exploitation.

Payoffs are also affected by a random perturbation, so the above-mentioned payoffs are just the expected values. A simple form such as the addition of $\epsilon \sim \mathcal{N}(0, \sigma^2)$ would be unsuitable when used in population simulations. As variance of independent random variables is additive, it would inevitably lead to an ever increasing dispersion of resource levels in the population. To avoid this issue, we adopted a perturbation in the form of a first-order autoregressive process that does not change either the mean or the variance of resources in the population (for a formal proof, see Bateson and Nettle 2017):

$$s_{t+1} = (1 - r) \cdot s_t + r \cdot \epsilon$$

$$\epsilon \sim \mathcal{N}(\mu, \frac{1 - r^2}{(1 - r)^2} \cdot \sigma^2)$$

Here, μ is the current mean resources in the population and σ^2 the population variance in resources. The term $(1-r) \in [0, 1]$ represents the desired correlation between an agent's current and subsequent resources, which leads to us describing r as the 'social mobility' of the population. The perturbation can be seen as a 'shuffle'. Each agent's resource level is attracted to μ with a strength depending on r, but this regression to the mean is exactly offset at the population level by the variance added by the perturbation, so that the overall distribution of resources is roughly unchanged. If $r = 1$, current resources are not informative about future resources.

1.1.2 The fitness function

The dynamic programming algorithm only requires a 'terminal reward' (the fitness function in the last time step T), then computes the fitness function at any earlier time t by backward induction. We make terminal reward a linear function of resource level s, but introduce a'desperation threshold'. During the computation, fitness is reduced by a fixed penalty ω for any resource value $s < 0$ at any time step.

1.1.3 The dynamic programming equation

Let I be the set of actions (*cooperate*,*exploit* and *hide*), which we shorten as $I = \{C, H, E\}$. For $i \in I$, we denote $\phi_t^i(s, \cdot)$ the probability density of resources in in time step t if, in time step $t-1$, the resource level is s and the chosen action i. The expressions of these functions were obtained through the law of total probability, conditioning on the possible outcomes of the actions (e.g. success or failure of the exploitation and of cooperation), and with the Gaussian density of the random variable ϵ.

We can now write the dynamic programming equation, which gives the backward recurrence relation to compute the fitness values (and the decisions) at the period t from the ones at the period $t+1$.

$$f_t(s) = \max_{i \in I} \mathbb{E}_i(f_{t+1})$$

$$= \max_{i \in I} \int (f_{t+1}(x) - \omega \cdot \mathbb{1}_{x<0}) \cdot \phi_{t+1}^i(s, x) dx$$

where we have noted $\mathbb{E}_i$ the conditional expectancy if action i is played. The optimal action for the resource state s and the period t is $\underset{i \in I}{\text{argmax}}\, \mathbb{E}_i(f_t)$.

The resource variable s was bounded in the interval $[-50, 50]$, and discretized with 1001 steps of size 0.1.

For any given set of parameters (summarised in table S1), we can therefore compute the optimal decision rule. Note that we can distinguish two types of parameters:

- 'Structural parameters', i.e. those defining the 'rules' of the game (the payoffs for the actions and the level of social mobility r, for example). In the subsequent simulation phase, these parameters will be fixed for any run of the simulations.

- 'Input parameters', such as p and s. In the simulation phase, these will evolve endogenously. In the computational of optimal policies, we seek optimal actions for all their possible values.

Optimal policies rapidly stabilize as the computation moves away from T. We report optimal actions at $t = 1$ as the globally optimal actions.

1.2 Population simulations

1.2.1 Simulation principles

We begin each simulation by initializing a population of $N = 500$ individuals, whose resource levels are randomly drawn from a Gaussian distribution with a given mean μ and variance σ^2. At each time step, interaction groups of n individuals are formed at random, and re-formed at each time step to avoid effects of assortment. There is no spatial structure in the populations. Each

Structural parameters

Symbol	Meaning	Typical value
r	Social mobility	0.1
x	Investment in cooperation	1
ω	Fitness cost imposed below the desperation threshold	5
α	Efficiency of cooperation	1.2
β	Benefit of successful exploitation	5
π	Punishment for exploitation	10
γ	Probability of punishment	1/3
n	Interaction group size	5
N	Population size	500
T	Total number of time steps	50

Inputs

Symbol	Meaning
μ	Population mean resources
σ	Population dispersion of resources (inequality)
p	Probability of being exploited (hence $1-p$ is trustworthiness)
s	Current level of resources

Functions

Symbol	Meaning
$f_t(\cdot)$	Fitness function at time step t
$\phi_t^i(\cdot, s)$	Probability density of resources at t for an agent whose resources are s in $t-1$ and playing action i

Table S1. Notation used in the model, and default values for structural parameters

individual always follows the optimal policy for its resources s and its estimate of p (see below).

To deal with the case where several members of the same interaction group choose to exploit, we choose one aat random that exploits, and the others are deemed to forage alone (in effect, there is nothing left for them to take). Also, when there is no cooperator in the group, all exploiters are deemed to forage alone.

Rather than providing each individual with perfect knowledge of the trustworthiness of the rest of the population $1-p$, we allow individuals to form an estimate (their *social trust*) from their experience. Social trust is derived in the following way.

- Each agent observes the decision of a sample of K individuals in the population, counts the number k of exploiters and infers an (unbiased) estimate of the prevalence of exploiters in the population: $k' = \frac{k}{K} \cdot N$ (rounded).

The size of the sample can be varied to alter the precision with which agents can estimate trustworthiness. Unless otherwise stated we used $K = 50$.

- Since p is the probability that there will be at least one exploiters in an interaction group, it is one minus the probability that there will be zero. Each agent computes this from k' by combinatorics:

$$\frac{\binom{N\cdot n-1-k'}{n-1}}{\binom{N\cdot n-1}{n-1}} \tag{1}$$

An intentional consequence of social trust being estimated through sampling is that there is some population heterogeneity in social trust, and therefore in decisions about which action to take, even for agents with the same resources s. Note also that agents infer trustworthiness not from observing the particular individuals in their current interaction group, but rather, from a cross-section of the entire population. Thus, the estimate is genuinely *social trust* (the perception that people in society generally do or do not behave well), rather than *personal trust* (reputational information about the specific individual with whom one is currently interacting).

2 Supplementary results

2.1 Effective long-term fitness function

Optimal policies are computed by backward induction starting from the final time step T, given the terminal reward function and the penalty ω. However, it is also possible to calculate the expected fitness at $t = 1$ for individuals with different levels of resources s, assuming that optimal policies are followed at every subsequent time step, and other parameters are held constant. This initial fitness-prospects function is shown in figure S1.

This function explains much of the behaviour of the model. Individuals beginning with very low resources (below the desperation threshold) have extremely low expected fitness, even following the optimal policy. This is because they are likely to incur the penalty for being in desperation in almost every subsequent time step. The gain in expected fitness from being immediately below to immediately above the desperation threshold is extremely steep; an individual who is immediately above can permanently avoid falling below, by either foraging alone, or, if the trustworthiness of the surrounding population is high enough, cooperating. Once individuals are well above the threshold, the marginal gain from resources being still higher is real but more modest; they will have higher resources at the terminal reward point, but this is much less influential than the fact of having avoided paying the penalty for desperation. This non-linear mapping explains conceptually why there is a combination of extreme risk-proneness in the vicinity of the threshold and below, and relative risk aversion well above it.

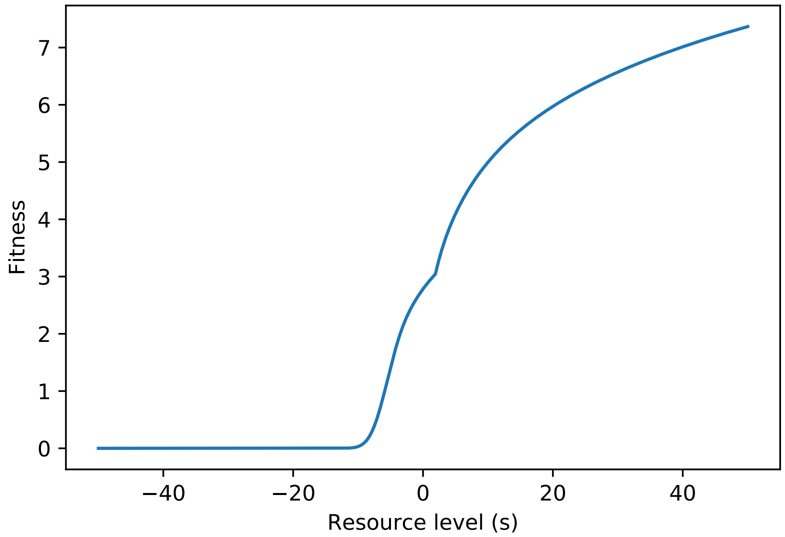

Figure S1. Expected fitness at $t = 1$ as a function of resources

2.2 Optimal choice between foraging alone and cooperating

For $s \gg 0$, considerations of risk-sensitivity become irrelevant, and the choice between foraging alone and cooperating is well approximated by choosing the action of the two with the higher expected return (since both actions involve the same investment). The expected return for foraging alone is x, whilst the expected return for cooperating is $(1-p)\alpha x$. Thus, the expected payoff for cooperating is superior when:

$$
\begin{aligned}
(1-p)\alpha x &> x \\
(1-p)\alpha &> 1 \\
(1-p) &> \frac{1}{\alpha}
\end{aligned}
$$

This inequality well captures the division between the foraging alone and cooperating zones shown in figure 1 of the main paper.

2.3 Cases where the expected payoff of exploitation is positive

In our default case, the expected payoff of exploitation is zero, and in the main paper we also consider cases where γ and/or π are larger and hence the expected payoff for exploitation is negative. We can also make the expected payoff for

exploitation positive, by making γ and/or π sufficiently small that $(1-\gamma)\beta > \gamma\pi$. These situations produce an optimal action policy where exploitation is favoured at either critically low, or comfortably high levels of resources (figure S3). In between, either cooperation or foraging alone is optimal, depending on trustworthiness. In these situations, those who are at a comfortable level of resources can afford to take the hit of a punishment, and thus can pursue risky but potentially profitable exploitation. It is individuals at an intermediate resource level, who are currently above the threshold but would be damagingly dragged below it by a punishment, who cannot risk exploiting.

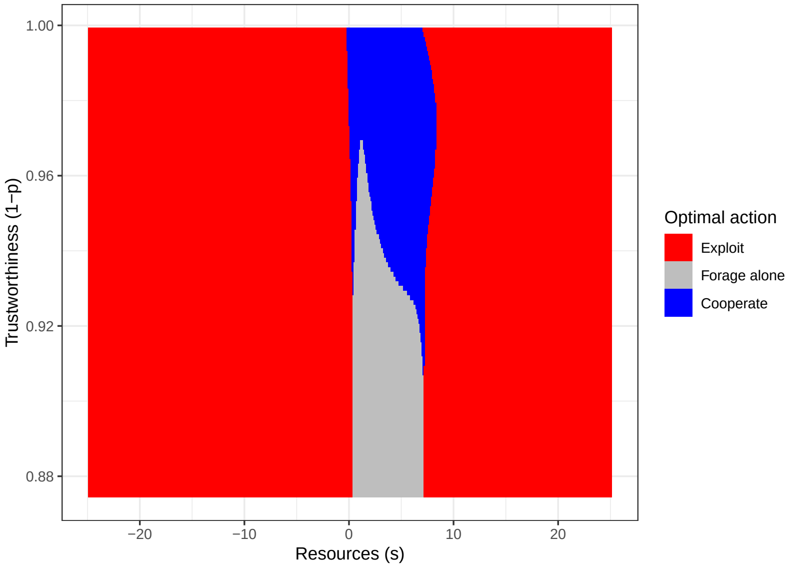

Figure S2. Optimal action policy with $\pi = 5$ and other parameters at their default values.

2.4 Implementation of exogeneous shock

We ran simulations with initial $\mu = 5.5$ and $\sigma = 4$ (other parameters have their default values), then switched to $\sigma = 3$ after 16 time steps (figure S4). As the figure shows, the response to the shock is slightly lagged. Over the course of a couple of time steps, the prevalence of exploitation reduces through the effects of the reduced inequality, but cooperation has not yet increased, since trust is still low. With exploitation rarer, social trust now increases one time step later, since social trust updates at the end of the time step. This then causes a cascade of switching to cooperation and the evolution of the virtuous circle.

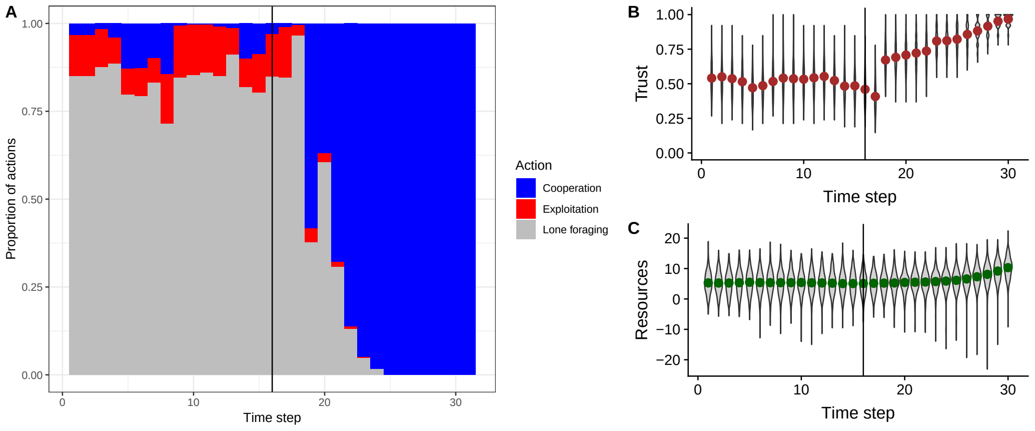

Figure S3. Phase transition provoked by exogeneous inequality reduction at time step 16. Parameter values from time step 1 are as figure 2A of main paper.

2.5 Varying interaction group size

The parameter n affects the simulations through the mapping between the number of exploiters in the population, and the probability of any particular cooperation group containing at least one exploiter (p), which, in turn, determines the agents' levels of social trust (estimates of $1 - p$). Concretely, for any given population prevalence of exploitation, increasing n will reduce the social trust of the individuals, as there is more likely to be at least one exploiter in a group of ten than in a group of five. This effect is plotted in figure S2.

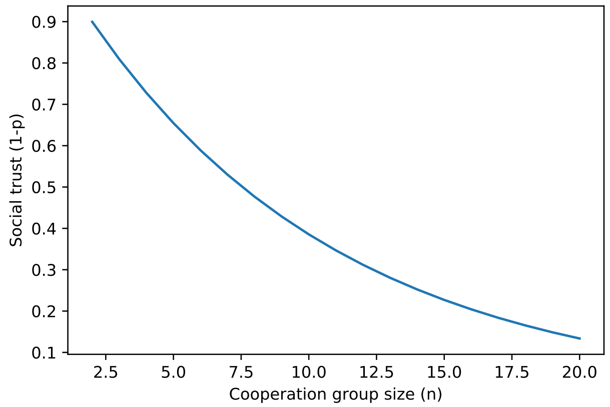

Figure S4. Impact of interaction group size on social trust

References

Bateson, Melissa and Daniel Nettle (2017). "The Telomere Lengthening Conundrum – It Could Be Biology". en. In: *Aging Cell* 16.2, pp. 312–319. ISSN: 1474-9726. DOI: 10.1111/acel.12555.

Houston, Alasdair I. and John M. McNamara (1999). *Models of Adaptive Behaviour: An Approach Based on State*. en. Cambridge University Press. ISBN: 978-0-521-65539-2.

Mangel, Marc and Colin Clark (1988). *Dynamic Modeling in Behavioral Ecology*. en. Princeton University Press. ISBN: 978-0-691-08506-7.
